# Nutrient Content Prediction and Geographical Origin Identification of Bananas by Combining Hyperspectral Imaging with Chemometrics

**DOI:** 10.3390/foods13223631

**Published:** 2024-11-14

**Authors:** Honghui Xiao, Chunlin Li, Mingyue Wang, Zhibo Huan, Hanyi Mei, Jing Nie, Karyne M. Rogers, Zhen Wu, Yuwei Yuan

**Affiliations:** 1 College of Food Science and Engineering, Ningbo University, Ningbo 315211, China; m15990294529@163.com; 2 Key Laboratory of Information Traceability for Agricultural Products, Ministry of Agriculture and Rural Affairs of China, Institute of Agro-Products Safety and Nutrition, Zhejiang Academy of Agricultural Sciences, Hangzhou 310021, China; chunlinli0304@163.com (C.L.); mhyaqq@163.com (H.M.); niejingnj@163.com (J.N.); 3 State Key Laboratory for Managing Biotic and Chemical Threats to the Quality and Safety of Agro-Products, Zhejiang Academy of Agricultural Sciences, Hangzhou 310021, China; 4 Analysis and Testing Center, Chinese Academy of Tropical Agricultural Sciences, Haikou 571101, China; hkwmy0815@163.com (M.W.); huanzhibo@163.com (Z.H.); 5 National Isotope Centre, GNS Science, 30 Gracefield Road, Lower Hutt 5040, New Zealand; k.rogers@gns.cri.nz

**Keywords:** banana, hyperspectral imaging, soluble solids content, potassium, origin discrimination, model

## Abstract

The nutritional quality of bananas and their geographical origin authenticity are very important for trade. There is an urgent need for rapid, non-destructive testing to improve the origin and quality assurance for importers, distributors, and consumers. In this study, 99 banana samples from a range of producing countries were collected. Hyperspectral data were combined with chemometric methods to construct quantitative and qualitative models for bananas, predicting soluble solids content (SSC), potassium content (K), and country of origin. A second derivative analysis combined with competitive adaptive weighted sampling (CARS) and random frog jumping (RF) was selected as the best pre-treatment method for the prediction of SSC and K content, respectively. Partial least squares (PLS) models achieved R^2^_p_ values of 0.8012 and 0.8606 for SSC and K content, respectively. Chinese domestic and imported bananas were classified with a prediction accuracy of 95.83% using partial least squares-discriminant analysis (PLS-DA) and an RF method that screened the spectral variables after a second pretreatment. These results showed that hyperspectral imaging technology could be effectively used to non-destructively predict the nutrient contents of bananas and identify their geographical origin. In the future, this technology can be applied to determine the nutritional quality composition and geographical origin of bananas from other countries.

## 1. Introduction

Banana (*Musa nana* Lour., from the genus Musa in the Plantain family) is a fruit abundant in carbohydrates, vitamins, minerals, flavonoids, and phenolic compounds [1]. At present, bananas are grown in over 130 countries worldwide, and most of them are cultivated in tropical and subtropical regions. In world trade, banana is positioned by the United Nations Food and Agriculture Organization as the fourth largest food crop after rice, wheat, and corn. The Philippines, India, China, and Ecuador are the world’s major banana producers, and they are key economic pillars for these countries. The annual global banana production in 2021 was 125 million tons, and the trade volume was USD 13.6 billion. During the same period, China’s banana production was 11.3 million tons, ranking second in the world, but China’s demand for bananas was greater than its production, and the demand gap was filled mainly by imports. There are certain differences in the quality, taste, nutritional value, and price of bananas from different countries, but it is hard to identify banana origin by appearance alone. A rapid, non-destructive origin verification method is urgently needed to improve consumer confidence around potential origin mislabeling and provide assurance for their preferred choice.

Banana is a nutrient-rich, aromatic, and tasty fruit that provides dietary energy and enhances immunity, benefiting human health. Banana soluble solids content (SSC) is an important index of ripeness and flavor quality [2]. Banana is also a high-calorie fruit that contains more trace potassium (K) than other fruits. Potassium is an important dietary element that not only contributes to and maintains the normal metabolism of cells but also maintains the excitability of neuromuscular tissues [3]. Therefore, measuring the SSC and K contents in bananas is vital.

In recent years, with the rapid development of sensors and data analysis technologies, non-destructive testing has been widely applied to determine the nutritional composition of apples [4], mango [5], peaches [6], kiwifruit [7], and other fruits. Currently, there is little literature on the use of rapid and non-destructive testing of SSC and K contents in bananas, especially for studies that use a novel combination of spectral features and imaging. Moreover, hyperspectral imaging technology can simultaneously obtain spatial scale and spectral information from objects while having the advantage of being fast, efficient, and non-destructive [8]. In the past, hyperspectral imaging has been successfully applied for fruit quality analysis and food safety detection. Li et al. [9] established a PLSR model based on plum soluble solids using hyperspectral imaging, and Furlanetto et al. [10] also used this technology to predict the potassium content in soybean leaves. Their results showed that specific nutrient contents could be quantitatively predicted with high accuracy (R^2^ of 0.88) during the entire development stage. Wang et al. [11] used hyperspectral imaging technology and an orthogonal partial least squares discriminant analysis (OPLS-DA) model to successfully classify red raspberries from three growing regions. These plant product studies demonstrated that it was potentially feasible to use hyperspectral imaging to predict the nutrient content of a banana and distinguish its origin.

In this paper, hyperspectral imaging data are used to establish the nutrient values of Chinese domestic and imported bananas. Quantitative and qualitative models were established to predict quality parameters and geographical origin, respectively. The results will provide a rapid and non-destructive approach to fruit quality supervision and further contribute to protecting consumer rights and interests.

## 2. Materials and Methods

### 2.1. Sample Preparation

**Figure 1:**
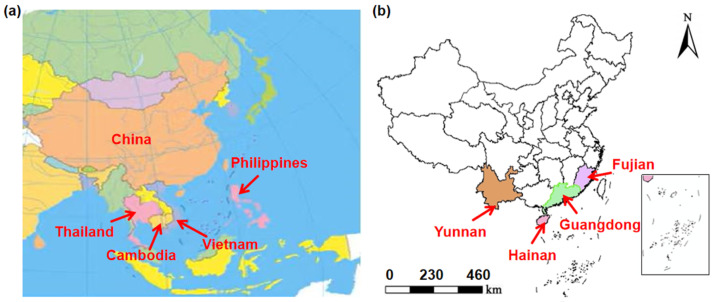
(**a**) Locations of bananas collected from other countries (n = 236), (**b**) locations of bananas collected from provincial China (n = 160).

In this study, 99 bunches of bananas were purchased from 32 fresh fruit supermarkets in Hangzhou, Zhejiang Province, with four bananas in each bunch, totaling 396 bananas. Among them, 59 bunches of bananas were from imported origins such as the Philippines, Vietnam, Cambodia, and Thailand (Figure 1a), and 40 bunches of bananas were domestically produced in Hainan, Yunnan, Fujian, and Guangdong provinces in China (Figure 1b), respectively.

Each banana bunch consisted of four bananas of similar size, color, and ripeness with yellow skins, similar appearance, and no obvious scarring damage. Bananas were stored at room temperature (24 to 26 °C) without any special treatment while undergoing analysis. Samples were numbered, and the hyperspectral data collection and physical and chemical analysis of banana samples were carried out within three days of purchase to ensure data consistency.

### 2.2. Nutrient Content Analysis

#### 2.2.1. Determination of SSC

The SSC of the bananas was determined by a handheld refractometer (TD32, Shanghai Precision Instruments Co., Ltd., Shanghai, China), which has a measurement range of 0–30 Brix and a resolution of 0.2 Brix. Four bananas were peeled, and each banana was divided into three equal parts. A portion of the mid-section from each banana was weighed and then combined to make a composite sample using a blender. The banana pulp was drained through a double layer of gauze until 1–2 drops of a clear exudate was recovered for soluble solids determination. Before determination, the Brix refractometer was calibrated with distilled water, the detection prism was wiped dry, and the exudate was applied [12]. Replicate analyses were averaged to eliminate experimental errors.

#### 2.2.2. Determination of Potassium Concentration

The remaining mid-sections of the four bananas were sliced into small pieces, combined to make a single sample, and placed into a vacuum freeze dryer (FD-A18N-50, Shanghai LNB Instrument Co., Ltd., Shanghai, China) at −30 ± 5 °C for 48 h. Once dried, the sample was ground into a fine, homogeneous power. Approximately 0.1 g of the powdered sample was accurately weighed into a digestion tube, into which 7 mL of nitric acid (≥65%) was added. The sample was sealed and allowed to stand for 2 h before being digested in a microwave digestion apparatus (TOPEX+, Shanghai Yiyao Instrument Technology Development Co., Ltd., Shanghai, China) at 200 °C for 30 min. Following digestion, the sample was cooled to room temperature; then, the acid was evaporated at 180 °C until no further fumes were given off. Subsequently, pure water was added to adjust the total volume to 25 mL. Then, a 0.45 μm needle with a filter was used to transfer the sample into a 15 mL centrifuge tube for analysis of the potassium content using an ICP-MS (8900ICP-QQQ, Agilent, Santa Clara, CA, USA). Kinetic energy identification (KED) the K concentration was also determined [13,14]. Internal standards of 50 μg/L Sc (GSB04-1757-2004) [13], Ge (GSB04-1728-2004) [13], Rh (GSB04-1746-2004) [13], and Re (GSB04-1745-2004) [13] were used as elemental abundance monitors and served to correct instrument drift. The recovery of the internal standard solution was 80–120%. Multi-point calibration utilized commercial elemental standard solutions obtained from the National Nonferrous Metals and Electronic Materials Analysis and Testing Center in Beijing. Reference materials (Cowpea-GBW10021 and Garlic-GBW10022) were obtained from the National Research Center for Reference Materials (Beijing, China) as laboratory quality control samples to evaluate the accuracy of the measurements.

### 2.3. Hyperspectral Image Collection

**Figure 2:**
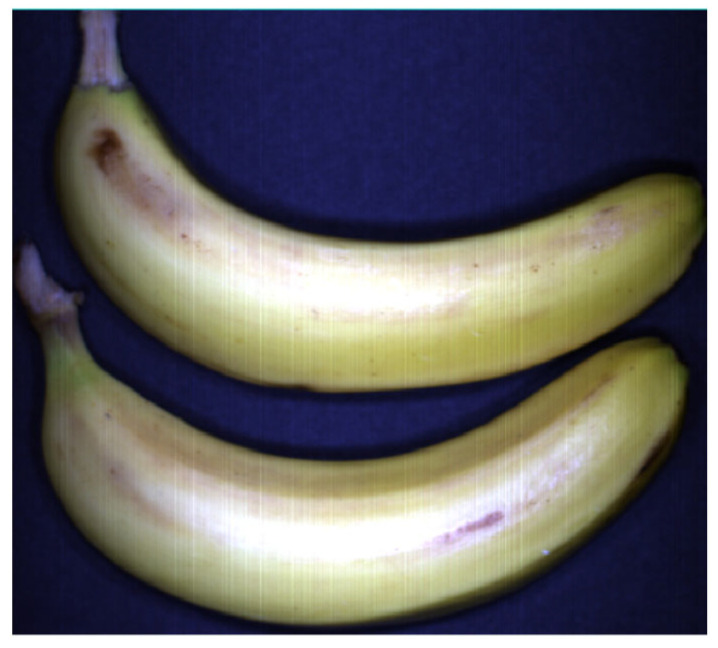
RGB image.

Spectral images of four unpeeled bananas were collected (prior to SSC and K content analysis) using a hyperspectral imager (Pika XC2, Resonon, Boston, MA, USA). The scanning mode of the instrument used linear push-broom scanning. The spectrum covered a wavelength range from 400 to 1000 nm, with 231 spectral channels, a hyperspectral camera that had a spatial resolution of 1600 pixels, and a maximum frame rate of 165 Hz. A banana was placed on a mobile platform directly under the spectral imaging device for hyperspectral analysis. The distance from the sample to the camera’s objective was fixed at 50 cm, the exposure time was 42.61 ms, the platform moving rate was 0.3802 cm·s^−1^, and the spectral resolution was 2.3 nm. The RGB images of banana samples collected by hyperspectral imaging technology are shown in Figure 2.

Before image acquisition, the instrument was preheated for 30 min. In order to reduce the effect of dark current and light source on the hyperspectral data, black and white correction was performed. A reference whiteboard was used to calibrate the system to obtain the white image information, and then the camera lens was covered to obtain the black image information. The calibrated image was used to extract spectral information, select characteristic wavelengths, and build relevant models [15].

### 2.4. Extraction of the Original Spectrum

To predict the SSC and K content in bananas and avoid differences in nutrient contents from different locations, the appropriate region of interest (ROI) was selected using a mid-section image of each banana sample. ENVI 5.6 software was used to process the acquired images and obtain an average spectrum of each sample. In this study, the averaged spectra were used for subsequent preprocessing. To eliminate other interference factors, such as noise, only spectral curves between 400 and 1000 nm were extracted.

### 2.5. Spectral Preprocessing Method

Six preprocessing methods were used to pretreat the acquired spectral data, namely, the multiple scatter correction algorithm (MSC) [16], standard normal variable transformation (SNV) [17], first derivative (1st) [18], second derivative (2nd) [19], centralized processing (Center), and savitzky-golay convolution smoothing (SG) methods [20]. Then, the preprocessed data were used for quality prediction, quantitative modeling, and origin discriminant classification modeling.

### 2.6. Modeling Method

Hyperspectral data were used to quantitatively predict the SSC and K content in bananas. The combined X-Y distance (SPXY) method was used to divide the sample set. The data were divided into 69 training sets and 30 prediction sets in a 2:1 ratio. A partial least squares (PLS) model was adopted as the modeling method for the quantitative prediction of SSC and K contents. The absorbance values were used for data inputs, and the contents of SSC and K were used as outputs. The optimal lv number was selected by cross-validation for the modeling process to avoid overfitting [21]. For the qualitative discrimination of banana origin, a PLS-DA model and a random forest (RFT) [22] model were used for geographical discrimination. The absorbance value of the spectral band was taken as the input; the Chinese domestic and imported banana origin classifications were used as the outputs. The principal component fraction was retrained by adjusting the parameters until the optimal model was obtained. The R^2^ (Coefficient of determination) and RMSE (Root mean square error) were used to evaluate the ability of the quantitative model to predict banana SSC and K contents. When the R^2^ was high (close to 1.0) and the RMSE was low, the model’s predictive origin classification accuracy was better. Classification accuracy is the most commonly used evaluation index in qualitative discriminant models, and a higher classification accuracy provides a more robust model.

### 2.7. Screening of Characteristic Wavelengths

Banana spectral data from 400 to 1000 nm were collected, and a total of 225 bands were obtained. When extracting the feature wavelengths, the 225 bands were taken as 225 variables. After establishing the full-band model, the competitive adaptive reweighted sampling algorithm (CARS) and random frog (RF) algorithms were used to extract the feature wavelengths of the best pre-processed spectral data. Finally, the quantitative and qualitative models were constructed, respectively, and the results were evaluated.

The CARS algorithm selected variable subsets through multiple sampling and then selected the smallest subset of RMSECV through interactive verification, using the best combination of variables [23]. In each sampling run, CARS performed four continuous processes, namely, Monte Carlo model sampling, forced variable deletion through an exponential decline function, ARS competitive variable deletion, and RMSECV calculation for each subset. In the experiment to predict the SSC and K contents of banana plants, the number of Monte Carlo simulations was set to 1000 and 500, the number of sampling times was 100 and 50, and 10-fold and 15-fold cross-validation methods were used, respectively. For testing the hyperspectral origin discrimination ability, the number of Monte Carlo simulations was set to 1000, the number of sampling times was set to 50, and an 8-fold cross-verification method was adopted.

The RF algorithm identified the most likely effective wavelengths by simulating a Markov chain characterized by normal distribution properties [24]. The RF algorithm calculated the probability of a variable being selected in each iteration. The importance of a variable was measured according to its probability, where, the higher the probability, the more important the variable. This process was divided into three steps: (1) randomly initialization of a variable subset V0 containing Q variables; (2) based on V0, a candidatevariable subset V* containing Q variables was proposed, where V* is accepted as V1 with a certain probability, and V0 is replaced by V1; (3) this step was repeated until N iterations were completed. Ultimately, the selection probability for each variable was calculated to gauge its importance [25]. Finally, 3 variables and 10 principal components were set for parameter initialization with 5000 iterations, while in the original discrimination model, the setup was initialized with 2 variables and 15 principal components until the termination of 10,000 iterations.

### 2.8. Software Processing

The hyperspectral image correction was carried out by the built-in software Spectron Pro. The reflectance data of the banana samples were extracted by ENVI software (ENVI 5.6), and the raw data preprocessing was performed by Unscrambler software (Unscrambler X10.1). The extraction of characteristic wavelengths and the establishment of models were performed by MATLAB software (MATLAB R2016a). The spectral curves and difference plots were generated by Origin software (Origin 2021). The difference analysis was carried out by SPSS software (IBM SPSS Statistics 25), using Excel software (Excel 2021) to summarize the SSC and K contents of the bananas.

## 3. Results and Discussion

### 3.1. Nutrient Quality Analysis

**Figure 3:**
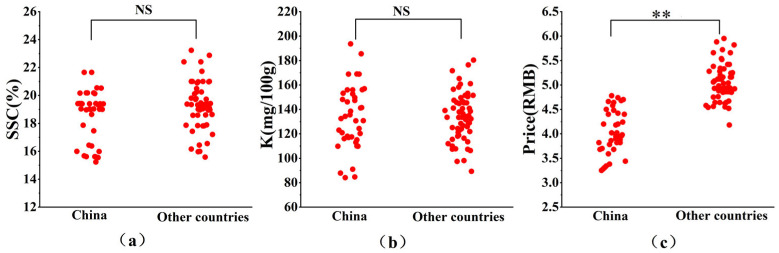
(**a**) SSC differences between Chinese domestic and imported bananas, (**b**) potassium differences between Chinese domestic and imported bananas, (**c**) price differences between Chinese domestic and imported bananas. Note: ** indicates significant differences. NS indicates no significant difference.

The measured SSC and K contents of bananas grown in different origins are shown in Table 1. The SSC of Chinese domestic and imported bananas ranged from 15.23 to 23.23% and were basically the same, with no significant differences between origins (Figure 3a). The K content of Chinese domestic and imported bananas ranged from 80 to 200 mg/100 g and was also similar for both Chinese domestic and imported bananas (Figure 3b). The average K content of Yunnan bananas was the highest in the study (161.72 mg/100 g), while the lowest average content was 123.42 mg/100 g in Cambodian bananas (Table 1). The range of K contents in bananas is not only related to banana varieties but also to soil, climate, and other factors [26,27]. In addition, a study by Suchitra et al. [28] found that the K content of bananas in Vietnam reached up to 629.27 to 921.35 mg/100 g. This high level may be caused by the application of potassium fertilizers often used in cultivation.

A notable price difference was seen between Chinese domestic and imported bananas (Figure 3c). The market price of imported bananas is significantly higher than Chinese domestically grown bananas, which may be related to the higher transportation costs, tariffs, and other expenses borne by importers.

### 3.2. Quantitative Prediction Model for Nutrients

#### 3.2.1. Preprocessing Method Selection

**Figure 4:**
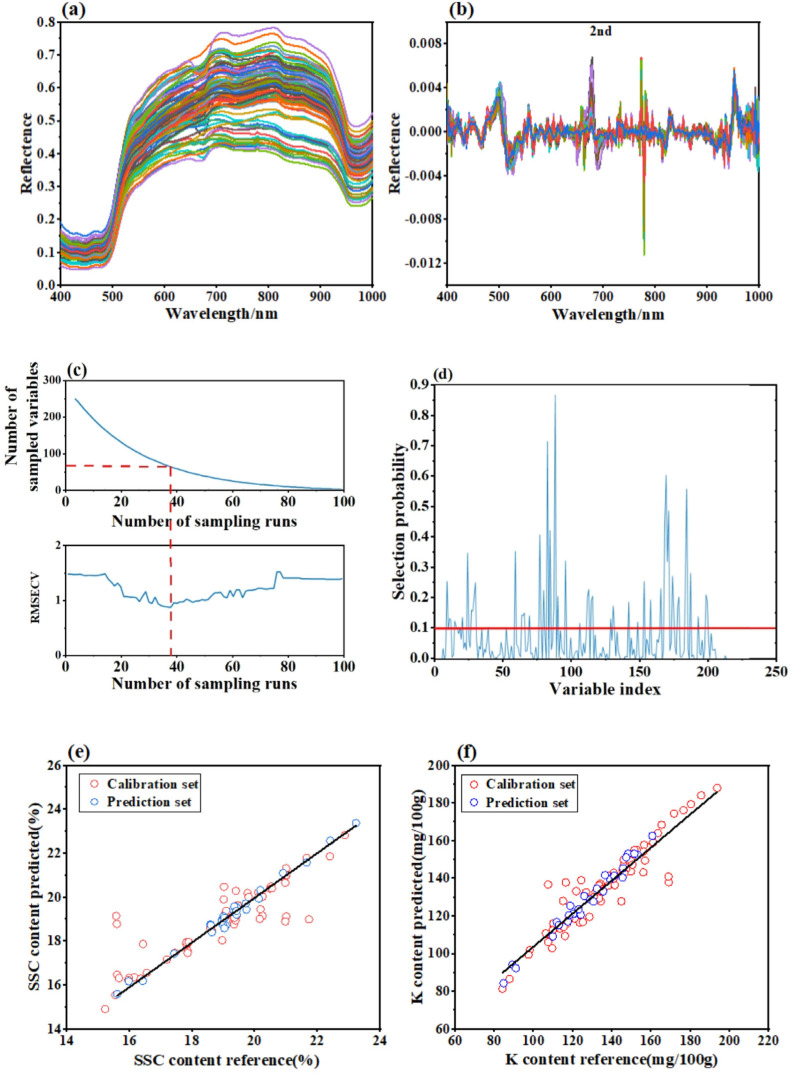
(**a**) Original spectral curves, (**b**) spectral curves after second derivative pretreatment, (**c**) characteristic wavelength selection process by CARS method when predicting SSC content, (**d**) characteristic wavelength selection process by RF method when predicting K content, (**e**) prediction results of SSC using spectral characteristics, and (**f**) prediction results of K content using spectral characteristics.

The extraction of spectrally valid information is a crucial task in spectral analysis. Usually, deviations from the spectral equipment itself, the influence of external light, and other analytical factors result in the collected spectral data having problems such as offset and nonlinear change [29]. Therefore, it is necessary to reduce the amount of redundant information unrelated to the sample information via various preprocessing methods to improve the accuracy of the model. The original spectral curves and pretreatment diagram of the banana samples are shown in Figure 4a. After preprocessing by the MSC and SNV methods, there was a significant reduction in the inter-sample variability induced by baseline drift and scattering in the spectral curves [30]. The first and second derivation methods (Figure 4b) enhanced the features and edge signals in the spectral data and removed noise and background interference [31]. The spectral curves became smoother after Center and SG processing [32]. After preprocessing, the PLS models for SSC and K contents were established, and the best spectral pretreatment method was selected.

A quantitative PLS model was established to predict the SSC. The second derivative pretreatment method had the best prediction effect, with R^2^_c_ and R^2^_P_ values of 0.7470 and 0.7137, respectively, and RMSEC and RMSEP values of 0.8738 and 0.9117, respectively (Table 2). A quantitative PLS model was established to predict K content. The second derivative pretreatment method also had a better predictive effect than the other pretreatment methods, with an R^2^_P_ of 0.7758 and an RMSEP of 0.9910. Due to the existence of redundant wavelength information in the spectra, when all the wavelength data were incorporated into the model, the model had a lower accuracy and reliability and required more calculation time. Therefore, a characteristic wavelength extraction method was used to select only the most representative wavelengths to improve the model’s accuracy.

#### 3.2.2. Extraction of Characteristic Wavelengths

The selection of characteristic wavelengths by the competitive adaptive reweighting algorithm (CARS) is based on Darwin’s survival of the fittest principle. The CARS method can be used to select specific wavelengths related to the target components and exclude irrelevant wavelengths or noisy spectra to reduce the amount of data processed and improve model performance. The predicted characteristic wavelengths of banana from SSC screening by the CARS method after the second derivative spectral pretreatment are shown in Figure 4c. The abscissa is the number of sampling runs, and the ordinate is the number of sampling variables and RMSECV. The number of sampling variables gradually decreases with increasing sampling time. The RMSECV decreases first and then increases. When the number of sampling runs reached 38, the RMSE reached a minimum, and the number of selected wavelength variables was 69. Therefore, 69 wavelength bands were effectively extracted from 225 bands, using only 30.67% of the initial wavelength data. CARS was applied to extract the characteristic wavelengths of the K content spectrum after 2nd derivative preprocessing. When the number of sampling runs reached 23, the RMSE reached a minimum, so 23 wavelength variables were selected.

The RF algorithm is a heuristic group optimization algorithm with the advantage of the reversible jumping Markov chain Monte Carlo algorithm, which is combined with the Monte Carlo algorithm to make the results of each screening random [33]. The RF algorithm was run 20 times, and the results are shown in Figure 4d. When predicting SSC content in bananas, the selection probability attained the highest number at the 172nd variable (the spectral band is 840.3 nm), the threshold was set as 0.1, and 43 variables with probabilities exceeding the threshold were selected as the characteristic bands. Figure 4d shows a schematic diagram of the characteristic wavelength extraction for predicting K content in bananas. The selection probability reached the highest value for the 92nd variable (the spectral band is 641.51 nm), the threshold was set to 0.1, and the 49 variables beyond the threshold were used as the best spectral feature bands.

#### 3.2.3. Modeling Results

After refining the characteristic wavelengths, the modeling results are shown in Table 2. A comparison of the different modeling treatments showed that the optimal model for predicting SSC in bananas was 2nd-CARS-PLS-DA, with R^2^_c_ and R^2^_P_ values of 0.8140 and 0.8012, respectively. The optimal model for predicting K content was 2nd-RF-PLS-DA, with R^2^_P_ and RMSEP values of 0.8606 and 0.8356, respectively. In previous studies, VIS/NIR spectroscopy was also used to establish a PLS model to predict the SSC of bananas, and the results showed that R^2^_P_ and RMSEP were 0.85 and 1.98, respectively [34]. However, the root-mean-square error of this study is smaller, which may be caused by a difference in sample numbers. Abenina et al. [35] used hyperspectral imaging and multivariate analysis to predict the potassium content of peach leaves, and the results showed that the established PLS model based on SNV preprocessing was the most effective, with an R^2^ of 0.8446.

As seen from the comparison of results in Table 2, CARS and RF algorithms used to predict the SSC greatly reduced the number of modeling calculations and significantly improved the modeling results compared with the full-band modeling. Therefore, the best pretreatment method in this study was the second derivative method. The CARS method was found to be more appropriate for variable selection in the banana SSC prediction model, while the RF method was best suited for variable selection in the banana potassium prediction model. Zhang et al. [36] also used the CARS algorithm to select characteristic variables when predicting SSC in oranges, but covariance between wavelengths still existed; therefore, a successive projection algorithm (SPA) was used to remove redundant wavelengths. A better model result was obtained, which could provide more accurate variables and results for the prediction of SSC and other nutrients.

Therefore, this study also provides a new way of thinking. When there is still wavelength redundancy in the selected feature bands, the optimal result can be obtained by re-screening. Azadnia et al. [37] used hyperspectral technology to predict K contents in apple leaves, and the results indicated that the RF algorithm was the best method for screening the characteristic wavelengths. This banana study obtained different results by establishing different models, which led to the use of different pretreatment methods, feature wavelength screening methods, and the use of different models to obtain the best results.

### 3.3. Geographical Origin Discrimination Model

In this study, five pretreatment methods and two models were used to classify banana samples from different regions and countries. The accuracy of these models is shown in Table 3. Compared with the results of the PLS-DA model established by five preprocessing methods, the accuracy of the results of the training set is improved, except in the case of the SNV method. After spectral pretreatment with the first, second, and SG methods, the accuracy of the prediction set was above 80%. It can be seen from the results in Table 3 that the optimal preprocessing method of the PLS-DA model was by using the second derivative and improved the accuracy by 10.63% and 8.33%, respectively, compared with the original data model. The random forest (RFT) model established by the second derivative preprocessing method had the best results, and the accuracy rate of the training set and prediction set were 86.67% and 80.00%, respectively. The overall accuracy of the PLS-DA model was clearly better than that of the random forest (RFT) model.

**Figure 5:**
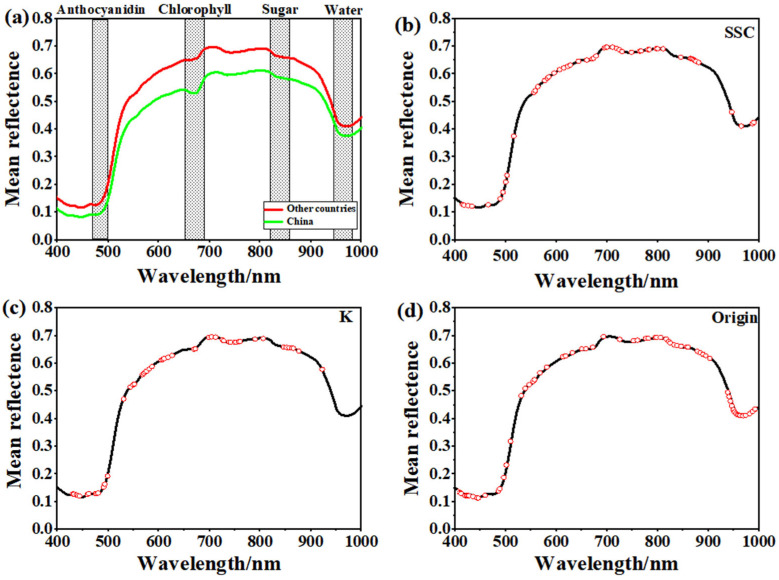
(**a**) Average reflectance spectrum of Chinese domestic and imported bananas, (**b**) characteristic wavelengths screened by the CARS method when predicting SSC, (**c**) characteristic wavelengths screened by the RF method when predicting K content, and (**d**) characteristic wavelengths screened during the identification of origin.

To select the best feature extraction method, CARS and RF algorithms were used to select the second derivative pretreatment spectra. The two methods were used to screen 49 and 78 characteristic bands, respectively. By comparing the number of feature bands extracted by the two methods, it was found that the number of feature bands screened by the RF method was significantly greater than that of the CARS method. Further observation of the feature band distribution showed that the feature bands screened by the RF method were evenly distributed, and all bands were involved (Figure 5d).

Characteristic wavelength variables screened by CARS and RF were imported into PLS-DA and random forest (RFT) models to obtain the classification results of banana samples. As shown in Table 3, the results of the PLS-DA model screened by characteristic variables were better than those established after optimal preprocessing. This suggests that both methods screen out a large amount of redundant information, retaining only important characteristic variables. The optimal model was established by using the random frog method to screen the characteristic variables, and the prediction set accuracy reached 95.83%. However, the accuracy of the RFT model established by the CARS method was lower. Therefore, 2nd-RF-PLS-DA was found to be the best origin classification model. Zhang et al. [38] used hyperspectral technology to distinguish apples from different origins, and the accuracy of the PLS-DA model established was more than 98%. Zhang et al. [39] and Sun et al. [40] also used hyperspectral technology to effectively distinguish yam and peach from different origins, respectively, indicating that spectral technology has made considerable progress and development in the field of food origin traceability.

### 3.4. Relationship Between Chemical Composition and Spectral Information

Due to a lack of prior hyperspectral information on bananas, the chemical composition of bananas was analyzed and compared using wavelengths from 400 to 1000 nm based on similar spectral reflectance data from other fruits. The average reflectance spectra of the extracted samples from Chinese domestic and imported bananas show that the spectral reflectance is relatively stable between 420 and 500 nm, with only small peaks and troughs. This region of stability may be related to the content of anthocyanins and carotenoids in bananas [41]. From 500 to 650 nm, the spectral reflectance increases with increasing wavelength, and, at 680 nm, an absorption peak appears, and the kurtosis changes, which may be related to the maturity of the bananas. With increasing ripeness, the chlorophyll content in bananas gradually decreases, and the carotenoid content gradually increases [42]. From 800 to 850 nm, the reflectance of the spectral curve decreases with increasing wavelength, which may be related to the sugar content of the bananas [43]. The absorption peak from 950 to 1000 nm may be related to the water content of the bananas [44].

Figure 5a. shows two average reflectance spectral curves of Chinese domestic and imported bananas. The shaded part is shown as the position related to chemical composition. The absorption peak at 800–850 nm in the spectra of the banana samples from other importing countries was slightly higher than the spectra of the Chinese domestic banana samples. According to Table 1, the mean SSC of the banana samples from other importing countries was slightly greater than the samples produced in China. The most important variable selected for SSC was found at 840.3 nm; therefore, it was again proven that there is a certain relationship between the hyperspectral band and the chemical composition of the fruit. Figure 5b–d shows schematic diagrams of the extracted characteristic wavelengths when predicting the SSC, K content, and origin discrimination, respectively. Wang et al. [45] discovered that the variations in the physical and chemical components as well as the color values of bananas were correlated with rainfall and temperature. Furthermore, it is likely that the hyperspectral information was probably also influenced by nutrient conditions. In the future, this technology may provide a valuable tool for regulatory authorities to identify the origin of bananas.

## 4. Conclusions

In this study, hyperspectral data from 99 banana bunches were collected, and second derivative order data were selected as the spectral data preprocessing method. The modeling effects of CARS and RF algorithms for the extraction of characteristic wavelengths were compared. The results show that the 2nd-CARS-PLS model and the 2nd-RF-PLS model can competently predict SSC and K contents in bananas. The 2nd-RF-PLS-DA model was established by using 78 eigen wavelengths screened by the RF method, which had the best classification effect, with accuracies of 95.83% for the prediction set. This demonstrates the potential use of hyperspectral imaging technology to identify different producing origins and nutritional quality of fruits to rapidly confirm food claims for agriculture and industrial applications.

## Figures and Tables

**Table 1:** The soluble solids contents (SSC) and K content of banana samples from different producing areas.

Producing Area		Sample Number	SSC (%)			K (mg/100 g)		
			Min	Max	Mean ± SD	Min	Max	Mean ± SD
China	Guangdong	2	19.00	20.13	19.5 ± 0.56 ^a^	130.64	130.70	130.67 ± 0.02 ^b^
	Fujian	4	16.37	19.42	18.4 ± 1.21 ^a^	141.44	152.63	147.37 ± 3.98 ^ab^
	Yunnan	4	17.46	19.40	18.8 ± 0.79 ^a^	118.05	193.66	161.7 ± 30.15 ^a^
	Hainan	30	15.23	21.65	18.5 ± 1.98 ^a^	84.13	168.98	128.5 ± 24.66 ^b^
Other countries	Thailand	4	17.86	22.40	20.5 ± 1.66 ^a^	115.42	139.03	126.0 ± 10.35 ^b^
	Cambodia	6	15.99	20.49	18.9 ± 1.55 ^a^	107.33	150.46	123.4 ± 19.18 ^b^
	Vietnam	17	15.58	23.23	19.0 ± 2.28 ^a^	106.23	176.43	139.6 ± 19.53 ^ab^
	Philippines	32	16.44	21.00	19.2 ± 1.09 ^a^	89.21	180.30	134.1 ± 19.39 ^ab^

Note: lowercase letters indicate a significant difference (*p* < 0.05) between producing areas with different letters.

**Table 2:** PLS model results with different spectral preprocessing methods and characteristic wavelength extraction methods.

Component	Method	Calibration Set		Prediction Set	
		R^2^_c_	RMSEC	R^2^_p_	RMSEP
Preprocessing method					
SSC	Raw	0.6495	1.0082	0.5918	1.1293
	MSC	0.5909	1.0214	0.5709	1.2745
	SNV	0.6215	1.0113	0.5742	1.2354
	1st	0.6680	0.9472	0.6280	1.1548
	2nd	0.7470	0.8738	0.7137	0.9117
	Center	0.5950	1.1195	0.5128	1.1676
K	Raw	0.7613	1.1433	0.7515	0.9797
	MSC	0.6900	1.1911	0.6393	1.4593
	SNV	0.6012	1.4704	0.5086	1.3523
	1st	0.7431	1.1152	0.7264	1.2028
	2nd	0.8078	1.0038	0.7758	0.9910
	Center	0.7262	1.1084	0.6847	1.3640
	SG	0.7085	1.1683	0.7018	1.2845
Feature wavelength extraction					
SSC	PLS	0.7470	0.8738	0.7137	0.9117
	CARS-PLS	0.8140	0.7432	0.8012	0.7958
	RF-PLS	0.7982	0.7667	0.7914	0.8326
K	PLS	0.8078	1.0038	0.7758	0.9910
	CARS-PLS	0.5269	1.0800	0.5129	1.2448
	RF-PLS	0.8753	0.7775	0.8606	0.8356

Note: MSC: Multiple scatter correction; SNV: Standard normal variable; 1st: First derivative; 2nd: Second derivative; Center: Centralized; SG: SavitZky-GoLay convolution smoothing; PLS: Partial least squares; CARS: Competitive adaptive reweighted sampling; RF: Random frog.

**Table 3:** Different model results using different spectral preprocessing methods and characteristic wavelength extraction methods.

Model	PLS-DA		RFT	
	Calibration Set	Prediction Set	Calibration Set	Prediction Set
Preprocessing method				
Raw	82.67	79.17	80.03	77.12
1st	92	87.5	81.08	80
2nd	93.3	87.5	86.67	80
MSC	82.67	75	81.08	76
SG	88	83.33	76	68
SNV	81.33	70.83	81.33	72
Feature wavelength extraction				
All wavelengths	93.3	87.5	86.67	80
CARS	96	91.67	82.67	76
RF	97.33	95.83	88	80

Note: 1st: First derivative; 2nd: Second derivative; MSC: Multiple scatter correction; SG: SavitZky-GoLay convolution smoothing; SNV: Standard normal variable; CARS: Competitive adaptive reweighted sampling; RF: Random frog.

## Data Availability

The original contributions presented in the study are included in the article, further inquiries can be directed to the corresponding authors.
